# Electronic Structure-, Phonon Spectrum-, and Effective Mass- Related Thermoelectric Properties of PdXSn (X = Zr, Hf) Half Heuslers

**DOI:** 10.3390/molecules27196567

**Published:** 2022-10-04

**Authors:** Bindu Rani, Aadil Fayaz Wani, Utkir Bahodirovich Sharopov, Lokanath Patra, Jaspal Singh, Atif Mossad Ali, A. F. Abd El-Rehim, Shakeel Ahmad Khandy, Shobhna Dhiman, Kulwinder Kaur

**Affiliations:** 1 Department of Applied Sciences (Physics), Punjab Engineering College (Deemed to be University), Chandigarh 160012, India; 2 Solar Thermal and Power Plants Laboratory, Physical-Technical Institute, Uzbekistan Academy of Sciences, Chingiz Aitmatov St., 2, Tashkent 100084, Uzbekistan; 3 Department of Mechanical Engineering, University of California, Santa Barbara, CA 93106, USA; 4 Department of Physics, Mata Sundri University Girls College, Mansa 151505, India; 5 Physics Department, Faculty of Science, King Khalid University, P.O. Box 9004, Abha 61413, Saudi Arabia; 6 Physics Department, Faculty of Science, Assiut University, Assiut 71516, Egypt; 7 Physics Department, Faculty of Education, Ain Shams University, Cairo 11771, Egypt; 8 ZJU-Hangzhou Global Scientific and Technological Innovation Center, School of Micro-Nano Electronics, Zhejiang University, Hangzhou 311200, China; 9 Department of Physics, Mehr Chand Mahajan DAV College for Women, Chandigarh 160036, India

**Keywords:** electronic structure, thermoelectric properties, phonon band structure, lattice thermal conductivity

## Abstract

We hereby discuss the thermoelectric properties of PdXSn(X = Zr, Hf) half Heuslers in relation to lattice thermal conductivity probed under effective mass (hole/electrons) calculations and deformation potential theory. In addition, we report the structural, electronic, mechanical, and lattice dynamics of these materials as well. Both alloys are indirect band gap semiconductors with a gap of 0.91 eV and 0.82 eV for PdZrSn and PdHfSn, respectively. Both half Heusler materials are mechanically and dynamically stable. The effective mass of electrons/holes is (0.13/1.23) for Zr-type and (0.12/1.12) for Hf-kind alloys, which is inversely proportional to the relaxation time and directly decides the electrical/thermal conductivity of these materials. At 300K, the magnitude of lattice thermal conductivity observed for PdZrSn is 15.16 W/mK and 9.53 W/mK for PdHfSn. The highest observed ZT value for PdZrSn and PdHfSn is 0.32 and 0.4, respectively.

## 1. Introduction

The ability of thermoelectric (**TE**) materials to convert heat to electrical energy has attracted a great deal of interest and can play a significant part in developing futuristic energy effective materials and devices [1]. Thermoelectric materials are eco-friendly, with no adverse effects on the environment, and are very important in daily life to achieve energy harvesting. The efficiency of **TE** material can be expressed by its figure of merit (**ZT**), which is defined as [2]

(1) $$\mathrm{ZT}=\frac{S^{2}\sigma T}{k}$$

where *S* is Seebeck coefficient, σ is electrical conductivity, T is absolute temperature, and k is total thermal conductivity. The total thermal conductivity (k) of crystal is sum of lattice thermal conductivity ($k_{L}$) and electronic thermal conductivity ($k_{el}$). All these parameters are related to each other, so it is difficult to alter the thermoelectric properties independently. 

Several techniques, such as electron-hole doping [3,4], strain engineering, forming a layered structures, effect of resonant levels [5], etc., have been used to enhance the value of ZT. The various efficient bulk thermoelectrics include Heusler materials [6,7,8,9], phonon glass and electron crystals (PGEC) [10,11], pentatellurides [12], clathrates [13], chalcogenides [14], skutterudite [15], oxides [16], and tin selenide [17], etc., which have low thermal conductivity and high electrical properties.

Here, we discuss the half Heusler (HH)-kind materials exhibiting an FCC structure in the form of XYZ [18] formulae units, where X and Y are the element of transition series of d-block, and Z is an element of group III–IV of p-block elements. In HH materials, X-atoms are positioned at (0, 0, 0), Y atoms at (0.25, 0.25, 0.25), and Z-atoms are located at (0.5, 0.5, 0.5). Half Heusler compounds have interesting properties due to their high value of Seebeck coefficient, power factor, and low thermal conductivity. MNiSn (M = Ti, Zr, Hf) are the most reliable half Heusler compounds due to their high ZT value, which is between the range from 0.7 to 1.5 [19]. XCoSb (X = Ti, Zr, Hf) compounds have gained attention because of their ZT value, which is equal to 1.0 at 1097 K with p-type doping [20,21]. K. Jia et al. found that CuLiX(X = Se, Te) are a good thermoelectric material due to their high ZT value, which is equal to 2.65 (1.7) for CuLiTe (CuLiSe) [22]. FeNbSb-based half Heusler compounds have also been explored, and their value of ZT is >1 [23,24]. M.K. Bamgbose investigated the thermoelectric properties of XIrSb (X = Ti, Zr, Hf) and found that ZT = 0.87, 0.95, and 0.90 for TiIrSb, TrIrSb, and HfIrSb at 800 K, respectively [25]. Fang et al. [26] have reported that due to large band degeneracy and low effective mass, the value of ZT = 1.5 at 1200 K for RuTaSb half Heusler. Thermoelectric properties of KBiX (X = Ba, Sr) were investigated by Z.F. Meghoufel et al. using ab initio principle, and they found the values of ZT = 2.68 and 1.56 for KBiBa and KBiSr, respectively [27]. R. Ahemad et al. reported ZT = 1 for all XMgN (X = Li, La, K) half Heusler materials [28]. Vikram and his co-workers explored the thermoelectric properties of Bi-based half Heusler alloys and reported ZT = 0.37 for HfRhBi, 0.42 for ZrIrBi, and 0.45 for ZrRhBi at 1300 K, respectively [29]. M. Zeeshan et al. investigated the thermal and electrical transport properties of two novel Fe-based Heusler alloys, namely FeTaSb and FeMnTiSb, and found ZT equal to 0.74 (FeNbSb), 0.72 (FeTaSb), and 0.46 (FeMnTiSb) at 1100 K [30]. ZrNiPb, ZrPtPb, and ZrPdPb have been reported with ZT values 1.71, 1.26, and 1.75, respectively [31]. J. Nagura and his co-workers explored the thermoelectric and mechanical properties of XHfSn (X = Ni, Pd, Pt) materials [32] using first principle calculations. Inspired by other half Heusler materials, we investigated the structural, electronic, mechanical, chemical, and thermoelectric properties of half Heusler PdXSn (X = Zr, Hf) in this paper using the first principle calculation and Boltzmann transport equation. To best out of our knowledge, the thermoelectric properties of PdZrSn Heusler material has not yet been explored, and also, there is no previous theoretical and experimental work reported on PdZrSn half Heusler material.

## 2. Computational Method

The calculations are performed within density functional theory (DFT) as implemented in Quantum Espresso code [33] (Version 6.7.0) using the norm-conserving pseudo potentials based on the Troullier Martins scheme [34]. The plane wave basis set and generalized gradient approximation (GGA) [35] with Perdew, Burke, and Ernzer of (PBE) exchange-correlation functional is used in this work. The plane-wave basis set is used to implement kinetic energy cutoff and charge density cutoff. The kinetic energy cutoff of 70 Ry and charge density cutoff of 700 Ry for PdHfSn and PdZrSn are used in calculations. Under the Monkhorst–Pack scheme [36] k-mesh 10 × 10 × 10 is used in the first irreducible Brillouin zone. 

The semi-classical Boltzmann transport theory, as implemented in the BoltzTraP code [37,38,39] and rigid band approximation (RBA), is used to determine the thermoelectric coefficient. For better convergence of results, highly dense k-points are used to calculate transport properties. The calculation of lattice thermal conductivity ($k_{L}$) is calculated using Slack’s equation [40,41,42], which is written as

(2) $$k_{L}=\frac{AM\theta_{D}^{3}V^{1/3}}{\gamma^{2}Tn^{2/3}}$$

Here, *M*, *V*, *n*, and γ are average atomic mass, volume, no. of atoms in the unit cell, and Gruneisen parameter, respectively. γ is calculated as

(3) $$\gamma =\frac{9-12{\left(\frac{v_{t}}{v_{l}}\right)}^{2}}{2+4{\left(\frac{v_{t}}{v_{l}}\right)}^{2}}$$

Here, $v_{l}$ is longitudinal, and $v_{t\;}$ is transverse velocity. The dimensionless constant *A* is computed as:

(4) $$A=\frac{2.43\times 10^{-8}}{1-\frac{0.514}{\;\gamma }+\frac{0.228}{{\left(\;\gamma \right)}^{2}}}$$

Deformation potential theory [43,44] based on effective mass approximation is used to determine the relaxation time (*τ*), which is calculated as

(5) $$\tau =\frac{8\pi^{1/2}{ћ}^{4}C_{ii}}{3{\left(m_{d}^{*}K_{B}T\right)}^{3/2}E_{d}^{2}}$$

where $m_{d}^{*}$ is the effective mass of DOS, *C_ii_* is elastic constant, and *E_d_* is deformation constant. In addition, *E_d_* is defined as

(6) $$E_{d}=\;\frac{\partial E_{edge}}{\partial \left(\frac{\Delta a}{a_{0}}\right)}$$

where $E_{edge}$ band energy corresponds to VBM and CBM for hole and electron, respectively, $a_{o}$ is the optimized lattice constant, and Δa is the distortion from equilibrium lattice parameter. The elastic constant (*C_ii_*) is estimated using the total energy with strain using a quadratic polynomial fit as

(7) $$C_{\mathrm{ii}}=\frac{1}{V_{o\;}}\frac{\partial^{2}E}{\partial {\left(\frac{\Delta a}{a_{o}}\right)}^{2}}$$

where $V_{o}$ is equilibrium volume. The details of the calculated values for this material are listed in Table 1.

## 3. Results and Discussion

### 3.1. Structure and Stability

**Figure 1:**
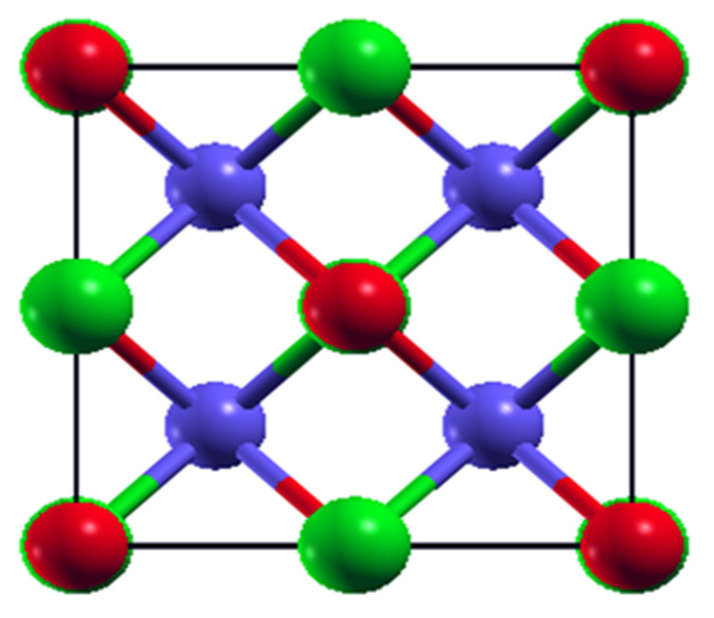
The crystal structure of PdXSn (X = Zr, Hf) compound. Blue, green, and red color represent Pd, X, and Sn atoms, respectively. The position occupied by Pd, X, and Sn are (0.25, 0.25, 0.25); (0.5, 0.5, 0.5); and (0, 0, 0), respectively.

Half Heusler materials PdXSn (X = Zr, Hf) have an FCC cubic crystal structure with space group F$\bar{4}$3 m symmetry as represented in Figure 1. The lattice constant of this series is calculated by GGA approximation corresponding to minimization of energy that is fitted by using the Birch–Murnaghan equation [45]. The optimized value of lattice parameters obtained for PdZrSn and PdHfSn are 6.41 Ǻ and 6.38 Ǻ, respectively, which are in accordance with previously published work as shown in Table 2.

The chemical stability of PdXSn (X = Zr, Hf) are examined with the help of formation energy ($\Delta E_{f})$ and cohesive energy $\left(\Delta E_{c}\right)$ calculations, using the following expressions [46,47]:

(8) $$\Delta E_{f}\;=\left[E{\left(\mathrm{PdXSn}\right)}_{n}^{Bulk}-nE{\left(\mathrm{Pd}\right)}^{bcc}-nE{\left(X\right)}^{bcc}-nE{\left(\mathrm{Sn}\right)}^{bcc}\right]/n$$

(9) $$\Delta E_{c}\;=\left[E{\left(\mathrm{PdXSn}\right)}_{n}^{Bulk}-nE{\left(\mathrm{Pd}\right)}^{atom}-nE{\left(X\right)}^{atom}-nE{\left(\mathrm{Sn}\right)}^{atom}\right]/2n$$

where $E{\left(\mathrm{PdXSn}\right)}_{n}^{Bulk}$ is *n* formula unit energy of PdXSn cell; $E{\left(\mathrm{Pd}\right)}^{bcc},\;E{\left(X\right)}^{bcc},\;E{\left(\mathrm{Sn}\right)}^{bcc}$ are energies of Pd, X, and Sn in stable structure, respectively. $E{\left(\mathrm{Pd}\right)}^{atom},\;E{\left(X\right)}^{atom},\;E{\left(\mathrm{Sn}\right)}^{atom}$ are energies of Pd, X, and Sn in free space. The formation energy (cohesive energy) of PdZrSn as −3.40 eV (−2.79 eV) and PdHfSn is −3.17 eV (−2.78 eV). The negative values of both formation and cohesive energies indicate that PdXSn(X = Zr, Hf) are chemically stable compounds and can be synthesized experimentally.

**Table 2:** Optimized lattice parameters of PdXSn (X = Zr, Hf) compound.

Parameter	Lattice Constant (Ǻ)	Band Gap (eV)
**PdZrSn**	6.41 (this work)	0.91 (this work)
	6.32 [48]	0.49 [48]
	6.321 [49]	0.43 [49]
	6.392 [50]	
**PdHfSn**	6.38 (this work)	0.82 (this work)
	6.354 [32]	0.40 [32]
	6.30 [48]	0.38 [48]

**Figure 2:**
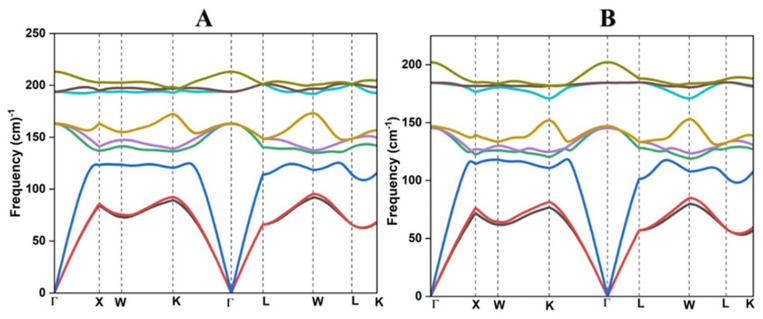
Phonon dispersion curve of (**A**) PdZrSn and (**B**) PdHfSn half Heusler materials.

The crystal structure of PdXSn (X = Zr, Hf) has been examined for dynamical stability with the help of phonon frequency calculations. For a dynamically stable system, the phonon frequency should be real and positive; a system with negative and imaginary frequency is not considered as dynamically stable. The phonon dispersion curve shown in Figure 2A,B indicates that there are no negative phonon frequencies that exist, which indicates the dynamical stability of both materials. In the phonon dispersion curve, there are three acoustical modes and six optical modes because these materials have three atoms in the primitive unit cell. The group velocity of phonon is described by the equation $v_{g}\;$= $\frac{d\omega }{dk}$, which represents the slope of related branches. The curvature of optical branches is flat, which corresponds to low group velocity, but the longitudinal acoustical branches having a linear variation seem to have a large group velocity and are primarily responsible for thermal conduction. Hence, the acoustical mode of phonon gives a large contribution to the lattice thermal conductivity of a material because the group velocity of this mode is very high.

### 3.2. Electronic Structure 

**Figure 3:**
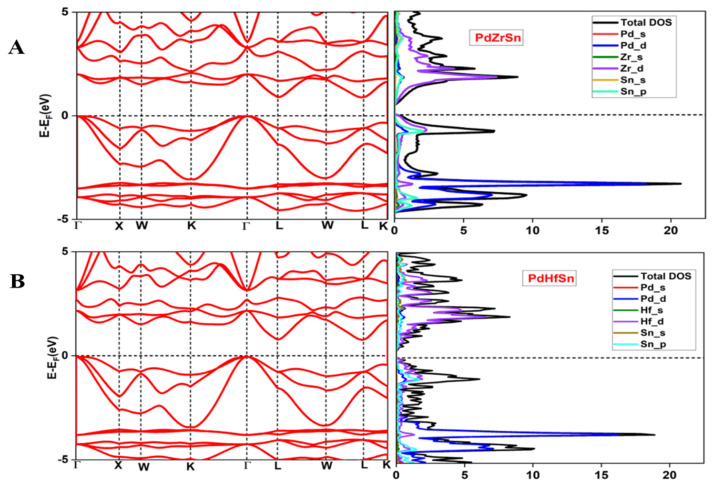
Electronic band structure and DOS for HH materials: (**A**) PdZrSn; (**B**) PdHfSn.

Figure 3 represents the band structure and density of states (DOS) of PdXSn (X = Zr, Hf), which are calculated at the optimized value of lattice constant using generalized gradient approximation. In-band structure calculations valence band maxima (VBM) are located at **Γ**-point, and conduction band maximum is located at L-point. Therefore, PdXSn (X = Zr, Hf) are indirect bandgap semiconductors with bandgaps of 0.91 eV and 0.82 eV, respectively. DOS shows atomic orbital’s contribution of the atoms. Near the fermi level, Zr-4d, Sn-5p orbitals for PdZrSn and Hf-5d, Sn-5p for PdHfSn have large contributions in the valence band and conduction band. Therefore, d-orbitals are expected to have a major role in determining the thermoelectric behavior of these HH materials. From the band structure, we observed that VBM are 3-fold degenerate, which consist of heavy and light bands. Heavy bands contribute to enhance the Seebeck coefficient, and light bands give a contribution to the charge carrier’s mobility. As a result, both types of bands enhance the TE performance of the materials.

Under the parabolic approximation, the effective mass for both types of charge carriers (electron and hole) is defined as

(10) $$m^{*}=\frac{{ћ}^{2}}{\frac{\partial^{2}E}{\partial k^{2}}}$$

This means that for a given *k*-point, the effective mass for a flat curvature will be higher in comparison to the effective mass for a sharp curvature. The effective mass of the density of states ($m_{d\;}^{*}$) is given by

(11) $$m_{d}^{*}=N_{V}^{2/3}(m_{x\;}^{*}.m_{y\;}^{*}.m_{z}^{*}{)}^{1/3}$$

$N_{V}$ is band degeneracy, and $m_{x}^{*}\;,m_{y\;}^{*},m_{z}^{*}$ are effective masses in the x, y, z directions, respectively. For an isotropic material, $m_{x}^{*}=m_{y\;}^{*}=m_{z}^{*}=m^{*}$; therefore, $m_{d}^{*}=N_{V}^{2/3}m^{*}\;$. The large value of *N_V_* and small value of m* give rise to the high value of Seebeck coefficient and carrier mobility.

**Figure 4:**
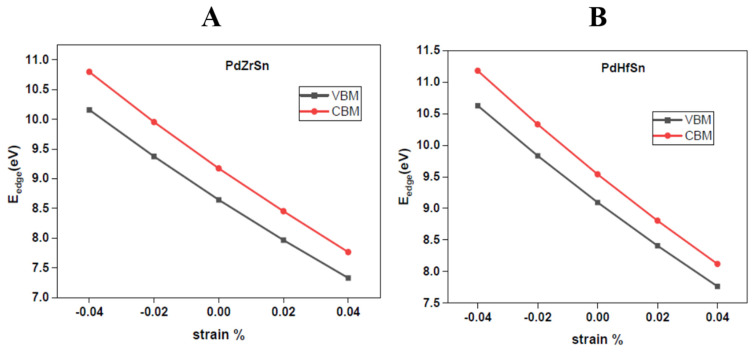
Variation of band edge energy with strain for PdZrSn (**A**) and PdHfSn (**B**).

The variation of band edge energy corresponding to valence band (VBM) and conduction band (CBM) with the applied strain is shown in Figure 4. The slope of the curves represents the value of the deformation constant (*E_d_*).

Born et al. [51] defined the following mechanical stability criteria using elastic parameters:

(12) $$C_{11}\;>\;0,\;C_{44}\;>\;0,\;\;C_{11}-\;C_{12}\;>\;0,\;\;C_{11}+2\;C_{12}\;>\;0$$

where $\;C_{11}$, $C_{12}$, and $C_{44}$ are elastic constants. The observed value of elastic constants of PdXSn (X = Ti, Hf, Zr) listed in Table 3 are found to satisfy the stability criteria, which implies that these HH compounds are mechanically stable. Using Voigt–Reuss–Hill approximations [52,53], the bulk modulus (*B*) and shear modulus (*G*) of PdXSn (X = Hf, Zr) were calculated. The bulk modulus (*B*), shear modulus (*G*), Young’s modulus, longitudinal ($v_{l}$), and transverse $\left(v_{t}\right)$ velocity is defined as

(13) $$B=\frac{\left(C_{11}+2C_{12}\right)}{3}$$

(14) $$G=\frac{\frac{C_{11}-C_{12}+C_{13}}{5}+\frac{5(C_{11}-C_{12})}{3\left(C_{11}-C_{12}+4C_{44}\right)}}{2}$$

(15) $$Y=\frac{9BG}{3B+G}$$

(16) $$v_{l}=\sqrt{\frac{G}{\rho }}$$

(17) $$v_{t\;}=\sqrt{\frac{\left(3B+4G\right)}{3\rho }}$$

Anderson’s formula [54] is be applied to compute the Debye temperature $\theta_{D}$ in terms of longitudinal and transverse velocity as

(18) $$\theta_{D}=\frac{ћ}{k_{B}}{\left(\frac{3n\rho N_{A}}{4\pi M}\right)}^{1/3}{\left[\frac{1}{3}\left(\frac{1}{v_{l}^{3}}+\frac{1}{v_{t}^{3}}\right)\right]}^{-1/3}$$

where ħ is reduced plank constant, $k_{B}$ is Boltzmann constant, n is no. of atom in the primitive unit cell, $N_{A}$ Avogadro’s number, and M is atomic mass of the unit cell. Cauchy’s pressure (*C*) and Pugh’s ratio [55] (*B/G*) are used for elaborating the brittleness and ductility of the material. The negative value of *C* = (*C*_12_ − *C*_44_) and *B/G* < 1.75, which implies the brittleness and vice-versa ductility of materials. The positive value of *C* = (*C*_12_ − *C*_44_) and *B/G* > 1.75 shows that both materials are ductile in nature.

### 3.3. Thermoelectric Properties 

**Figure 5:**
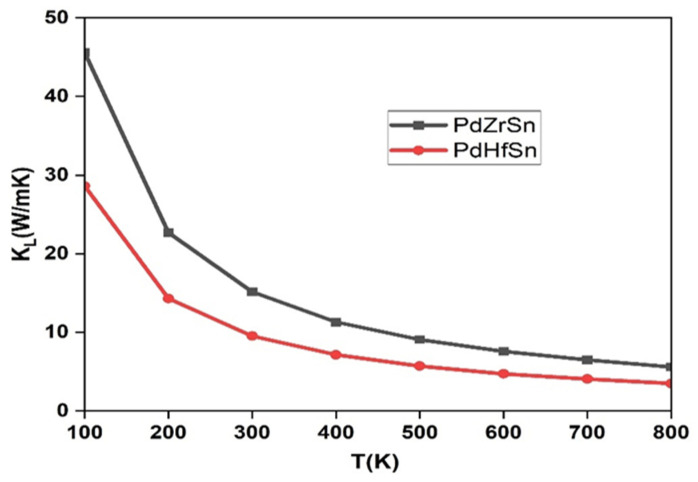
Variation of lattice thermal conductivity with temperature for PdZrSn and PdHfSn.

The thermoelectric parameters were calculated to find out the thermoelectric performance of PdXSn (X = Zr, Hf) at various temperatures using the Boltzmann transport equation. Figure 5 shows the variation of lattice thermal conductivity with temperature, and the value of lattice thermal conductivity is 15.16 (9.53) W/mK for PdZrSn (PdHfSn) at 300 K. The lattice thermal conductivity decreases with increase in temperature due to lattice scattering.

**Figure 6:**
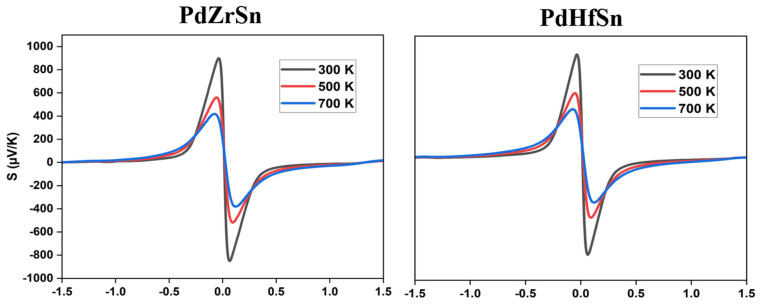

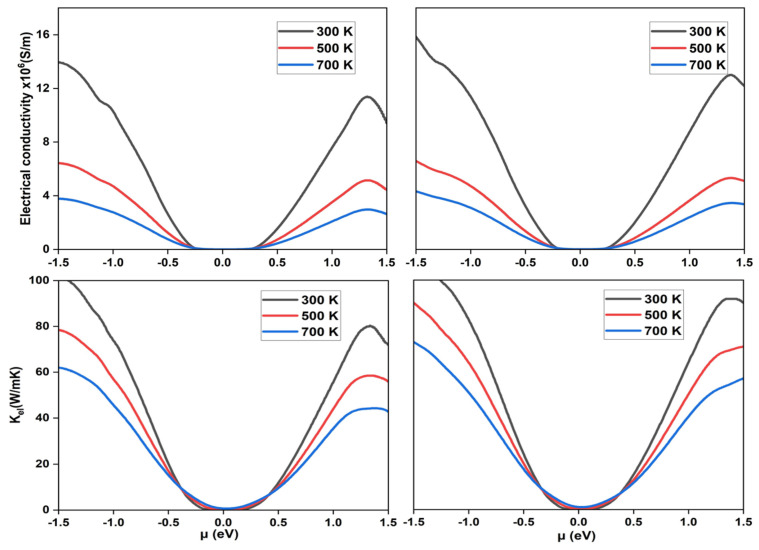
Seebeck coefficient and electrical conductivity and electronic part of thermal conductivity ($k_{el}$) for both PdZrSn (**left** panels) and PdHfSn (**right** panels) materials at various temperature (300 K, 500 K, 700 K) as a function of chemical potential (µ).

Figure 6 represents the Seebeck coefficient (*S*), electrical conductivity (σ), and electronic thermal conductivity ($k_{el}$) for both PdZrSn and PdHfSn materials at various temperatures (300 K, 500 K, 700 K). According to Mott’s formula [27], the Seebeck coefficient is directly proportional to DOS effective mass and temperature but inversely proportional to carrier concentration. It is given by

(19) $$S=\frac{8\pi^{2}k_{B}^{2}}{3eh^{2}}m_{d}^{*}T{\left[\frac{\pi }{3n}\right]}^{2/3}$$

Seebeck coefficient decreases as the carrier concentration increase with temperature. The calculated band structure for these materials features a sharp conduction band and a flat valence band, indicating high transport characteristics. The highest value of the Seebeck coefficient at room temperature is 900 µV/K and 763 µV/K for PdZrSn and PdHfSn, respectively. 

For calculation of total thermal conductivity, we have to evaluate the electronic thermal conductivity ($k_{el}$). The value of $k_{el}$ and σ are in the form of $\frac{\sigma \;}{\tau }$ and $\frac{k_{el}}{\tau }$, where τ is relaxation time. Electrical conductivity is inversely proportional to effective mass and depends directly upon relaxation time. At room temperature, the obtained value of electrical conductivity for PdHfSn (~17.46 × 10^6^ S/m) is larger than PdZrSn (~13.96 × 10^6^ S/m), and it decreases exponentially due to thermal collisions when temperature is increased. The temperature behavior of $k_{el}$ is similar to σ because both types of conductivity decrease with increase in temperature. It is observed that the largest value of electronic thermal conductivity is 101.88 W/mK and 127.08 W/mK for PdZrSn and PdHfSn, respectively, at room temperature. 

**Figure 7:**
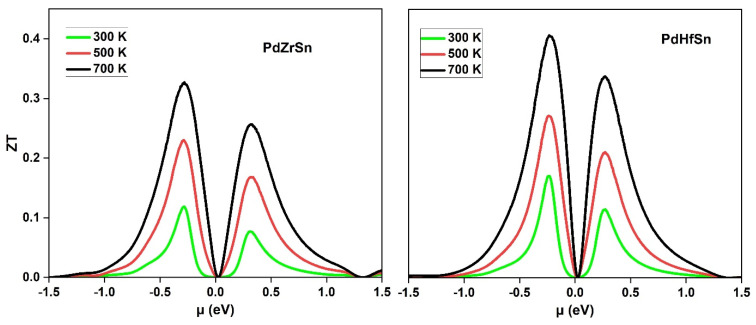
Variation of Figure of merit (ZT) of PdZrSn and PdHfSn materials with chemical potential at various temperatures.

The Seebeck coefficient, electrical conductivity, and total thermal conductivity are used to determine the dimensionless figure of merit ZT. Figure 7 shows the ZT value as a function of chemical potential for PdZrSn and PdHfSn materials. ZT gradually varies with temperature and attains the highest value 0.32 for PdZrSn and 0.4 for PdHfSn at 700 K. The comparison of ZT with other half Heusler compounds are given in Table 4. Apart from half Heusler compounds, the calculated ZT value of PdZrSn and PdHfSn is greater than or comparable with other materials such as quaternary Heusler compounds CoZrMnX (X = Al, Ga, Ge, In) [56], FeRhCrX (X = Si, Ge) [57], LiTiCoX (X = Si, Ge) [58] with ZT~(0.02–0.14), full Heusler Fe_2_ScX (X = P, As, Sb) [59] with ZT~(0.2–0.52), and SrTiO_3_ [60] with ZT = 0.07.

## 4. Conclusions

The thermoelectric properties of half Heusler compounds PdZrSn and PdHfSn have been studied under the perspective of density functional theory. PdZrSn and PdHfSn are indirect bandgap semiconductors with a bandgap of 0.91 eV and 0.82 eV, respectively. These materials are mechanically, chemically, and dynamically stable. Seebeck coefficient, electrical conductivity, total thermal conductivity, and ZT value are calculated at various temperatures with respect to chemical potential. Pugh’s ratio shows the ductile nature of both PdZrSn and PdHfSn. The highest value of ZT for PdZrSn is 0.32 and PdHfSn is 0.4 for p-type doping. The ZT value of PdHfSn is higher than PdZrSn. However, ZT values shows that both PdZrSn and PdHfSn are good for thermoelectric performance and certainly give guidance for experimental work. 

## Figures and Tables

**Table 1:** DOS effective masses ($m_{d}^{*}$), deformation constant (*E_d_*), elastic constant (*C_ii_*), and relaxation time (τ) of PdZrSn and PdHfSn at 300 K.

Compound	Electrons				Holes			
	$m_{d}^{*}\;(m_{o})$	*E_d_* (eV)	*C_ii_* (GPa)	τ (fs)	$m_{d}^{*}\;(m_{o})$	*E_d_* (eV)	*C_ii_* (GPa)	τ (fs)
PdZrSn	0.13	37.88	166.7	0.690	1.28	35.33	166.7	0.026
PdHfSn	0.128	38.21	163	0.698	1.12	35.74	163	0.030

**Table 3:** Elastic constants ($\;C_{11}$, $C_{12}$, and $C_{44}$ ), density (ρ), longitudinal velocity ($v_{l}$), transverse velocity ($v_{t}$), bulk modulus (*B*), shear modulus (*G*), Debye temperature ($\theta_{D}$), and Pugh’s ratio (*B/G*) for PdZrSn and PdHfSn compounds.

Property	PdZrSn (This Work)	PdHfSn (This Work)	(PdHfSn) Others 26
*C*_11_ (GPa)	178.8	175.1	179.5
*C*_12_ (GPa)	96.6	90.4	88.6
*C*_44_ (GPa)	76.7	79.0	69.6
ρ (gcm^−3^)	7.96	10.29	-
$v_{l}$ ** (ms^−1^)**	5055.9	4417.7	-
$v_{t\;}$ ** (ms^−1^)**	2738.0	2445.7	-
*B* (GPa)	124.0	118.6	117.0
*G* (GPa)	59.7	61.7	59.5
$\theta_{D}$ (K)	322.5	288.6	-
Pugh’s ratio (*B/G*)	2.07	1.92	-

**Table 4:** Comparison of Seebeck coefficient (*S*), electrical conductivity (σ), and thermal conductivity ($k_{L}$) (at 300 K) and maximum ZT of PdXSn (X = Zr, Hf) with other half Heusler materials.

Compound	*S* (µV/K)	σ (S/m)	$k_{L}$ (W/mK)	ZT Value
PdZrSn (our work)	900	13.96 × 10^6^	15.16	0.32
PdHfSn (our work)	763	17.46 × 10^6^	9.53	0.40
PtZrSn [47]	1533	4.00 × 10^5^	16.96	0.24
PtHfSn [47]	1649	6.42 × 10^5^	10.04	0.57
HfRhSb [61]	252	1.5 × 10^5^	17.35	0.42
ZrNiSn [62]	275	4 × 10^5^	7.00	0.64
ZrIrBi	255.9	26.5× 10^4^	2.00	0.42
ZrRhBi	319.8	8.7× 10^4^	-	0.43
HfPtSn [63]	196	5.2	14.90	0.05

## Data Availability

The data presented in this study are available on request from the corresponding author.
